# Structure and folding of four putative kink turns identified in structured RNA species in a test of structural prediction rules

**DOI:** 10.1093/nar/gkab333

**Published:** 2021-05-12

**Authors:** Lin Huang, Xinli Liao, Mengxiao Li, Jia Wang, Xuemei Peng, Timothy J Wilson, David M J Lilley

**Affiliations:** Guangdong Provincial Key Laboratory of Malignant Tumor Epigenetics and Gene Regulation, Sun Yat-Sen Memorial Hospital, Sun Yat-Sen University, Guangzhou 510120, PR China; Cancer Research UK Nucleic Acid Structure Research Group, MSI/WTB Complex, The University of Dundee, Dow Street, Dundee DD1 5EH, UK; Department of Chemistry, College of Chemistry and Chemical Engineering, Xiamen University, Xiamen 361005, PR China; Guangdong Provincial Key Laboratory of Malignant Tumor Epigenetics and Gene Regulation, Sun Yat-Sen Memorial Hospital, Sun Yat-Sen University, Guangzhou 510120, PR China; Cancer Research UK Nucleic Acid Structure Research Group, MSI/WTB Complex, The University of Dundee, Dow Street, Dundee DD1 5EH, UK; Guangdong Provincial Key Laboratory of Malignant Tumor Epigenetics and Gene Regulation, Sun Yat-Sen Memorial Hospital, Sun Yat-Sen University, Guangzhou 510120, PR China; Cancer Research UK Nucleic Acid Structure Research Group, MSI/WTB Complex, The University of Dundee, Dow Street, Dundee DD1 5EH, UK; Cancer Research UK Nucleic Acid Structure Research Group, MSI/WTB Complex, The University of Dundee, Dow Street, Dundee DD1 5EH, UK

## Abstract

k-Turns are widespread key architectural elements that occur in many classes of RNA molecules. We have shown previously that their folding properties (whether or not they fold into their tightly kinked structure on addition of metal ions) and conformation depend on their local sequence, and we have elucidated a series of rules for prediction of these properties from sequence. In this work, we have expanded the rules for prediction of folding properties, and then applied the full set to predict the folding and conformation of four probable k-turns we have identified amongst 224 structured RNA species found in bacterial intergenenic regions by the Breaker lab (1). We have analyzed the ion-dependence of folding of the four k-turns using fluorescence resonance energy transfer, and determined the conformation of two of them using X-ray crystallography. We find that the experimental data fully conform to both the predicted folding and conformational properties. We conclude that our folding rules are robust, and can be applied to new k-turns of unknown characteristics with confidence.

## INTRODUCTION

The majority of RNA sequences transcribed in cells do not code for protein, and they have diverse functions. Many of these regulate gene expression in various ways, and riboregulation is important in all domains of life (2,3). In humans, long non-coding RNA species regulate physiological processes (4), and are implicated in disease (5). Thus they also offer potential therapeutic targets. However, for the great majority of these species, only nucleotide sequence information is available, and 3D structures have been determined for very few of them. Moreover their folding properties are largely unknown. Computational approaches to predict the structures of RNA molecules based upon sequence therefore assume considerable importance (6–8). But where possible predictive methods should be informed by experimental data.

**Figure 1.**
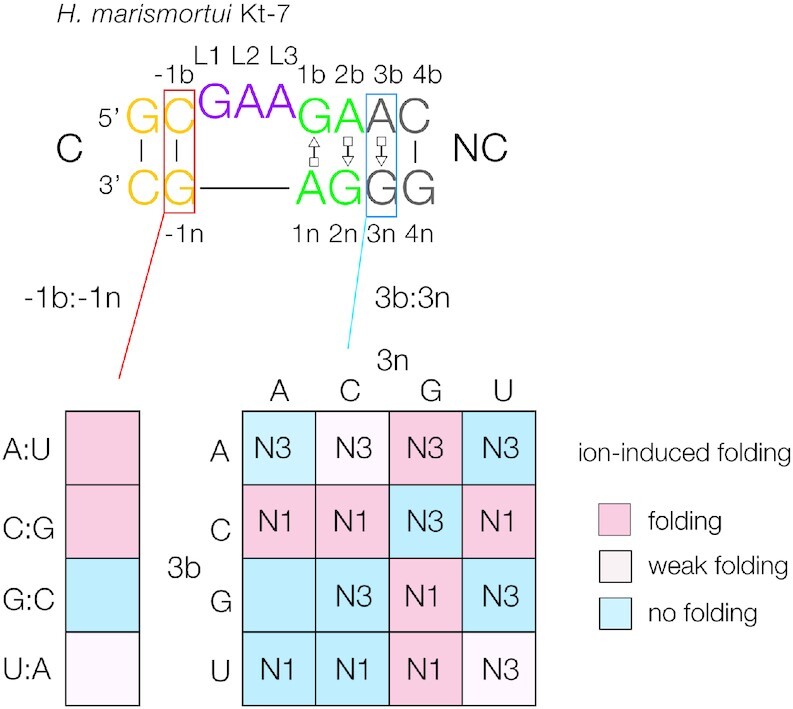
Summary of the rules for the effects of local sequence on the ion-induced folding and the N3/N1 conformation of k-turns. The sequence of the standard k-turn *H. marismortui* Kt-7 is shown at the top, with the nucleotide nomenclature shown, and our standard coloring scheme used throughout this paper. The helices are named C (canonical) and NC (non-canonical). Nucleotide positions are identified using the scheme given in Liu and Lilley (14), where nucleotides in the NC-helix are numbered positively out from the loop, while nucleotides in the C-helix are numbered negatively out from the loop. Bulged strand nucleotides have the suffix *b*, while the lower strand nucleotides have suffix *n*. Loop nucleotides are numbered sequentially 5′ to 3′, with the suffix L. The arrays are colored by the conferred folding properties; sequences that fold well on addition of Mg^2+^ ions are shown pink, those with weak folding in light pink and those that do not fold in Mg^2+^ ions are shown blue. The folding properties of the k-turns with the four possible Watson-Crick base pairs in the −1b:−1n position are shown with the same color scheme; two have been determined in this work. The N3/N1 conformations are determined by the 3b:3n sequences; these are written into the 4 × 4 array. The *trans* Hoogsteen:sugar conformations of the A:G base pairs are shown using the Leontis−Westhof notation (37).

To a first approximation, RNA secondary structure can be reduced to rigid helical sections that are connected by junctions, the conformations of which will determine the trajectory of the helices. These will set up the formation of longer-range tertiary interactions that generate the overall three-dimensional fold. The kink-turn (k-turn) is a widespread helical junction in RNA. The standard k-turn comprises a duplex RNA containing a three-nucleotide bulge followed by tandem G:A and A:G base pairs (9–11) (Figure 1). The G:A base pairs adopt *trans* sugar-Hoogsteen conformation, also known as sheared base pairs, and the overall structure is tightly kinked, with an included angle between the axes of the flanking helices of 50°. The conformation of the k-turn is determined by two properties;

1. In the absence of metal ions or binding proteins, the RNA adopts a conformation that is not tightly kinked, but rather bent in the manner of a simple three-nucleotide bulge. Many (but not all, see below) k-turns adopt the k-turn conformation on addition of divalent metal ions, but some will not. The great majority of k-turns will fold on binding of proteins such as the L7Ae family, including those that do not undergo folding in metal ions (12).
2. The conserved adenine nucleobases of the sheared G:A base pairs both form important cross-strand hydrogen bonds with 2′-hydroxyl groups at the L1 and −1n positions (13,14). However the bond donated by −1n O2′ can be accepted by either A2b N3 or N1 (15,16) (the nomenclature of nucleotide positions in the k-turn is summarized in Figure 1). This affects the G2n:A2b base pair structure, and results in a rotation of the C-helix with a potential effect on any tertiary interactions mediated by the k-turn (15). The known k-turns divide into two groups of approximately equal size depending on their N3 or N1 conformation.

We have explored what sequences determine whether or not a given k-turn sequence folds in response to metal ions (12,17), and whether it adopts the N3 or N1 conformation (18). The results can be summarized by a series of folding and conformation rules (Figure 1). The 3b:3n sequence has a major influence on both properties, and the folding characteristics can also be affected by the −1b:−1n sequence. The 3b:3n position can be any of the 16 possible pairings, and this is a strong determinant of folding ability in metal ions. Experimentally we discovered three rules (12):

1. 3b:3n = Watson−Crick base pairs lead to poor or non-existent folding in metal ions.
2. 3b = C leads to good folding in response to metal ions.
3. k-turns with 3n = G also fold well in response to metal ions.

In addition, rule 1 takes precedence over rules 2 and 3. The 3n = G rule was explained by the formation of well-ordered divalent metal ions that bound in the major groove (12). The −1b:−1n sequence is also known to influence ion-induced folding. In many k-turns −1b:−1n = C:G, but inversion to −1b:−1n = G:C led to highly impaired folding (17). A sub-set of human boxC/D snoRNAs have −1n = C. This creates a target for the METTL3-METTL14 methyl transferase (19), and half of these k-turns are known to undergo methylation at N^6^ of adenine 1n, thereby preventing the k-turn from folding as the first step of the snoRNP assembly (20). In this work we explore the effect of variations at the −1b:−1n position to include all four Watson−Crick base pairs.

We have found that the 3b:3n sequence also determines whether the N3 or N1 conformations are adopted in the folded k-turn (18), and we have assigned the conformation associated with 15/16 potential 3b:3n sequences (11). We have shown how the folding and conformational rules can rationalize the distribution of k-turn sequences in a number of groups of natural k-turns. They also have predictive power for sequences of unknown structure and function, and in this work we have applied our rules to putative k-turns identified in a set of new RNA sequences recently identified.

**Figure 2.**
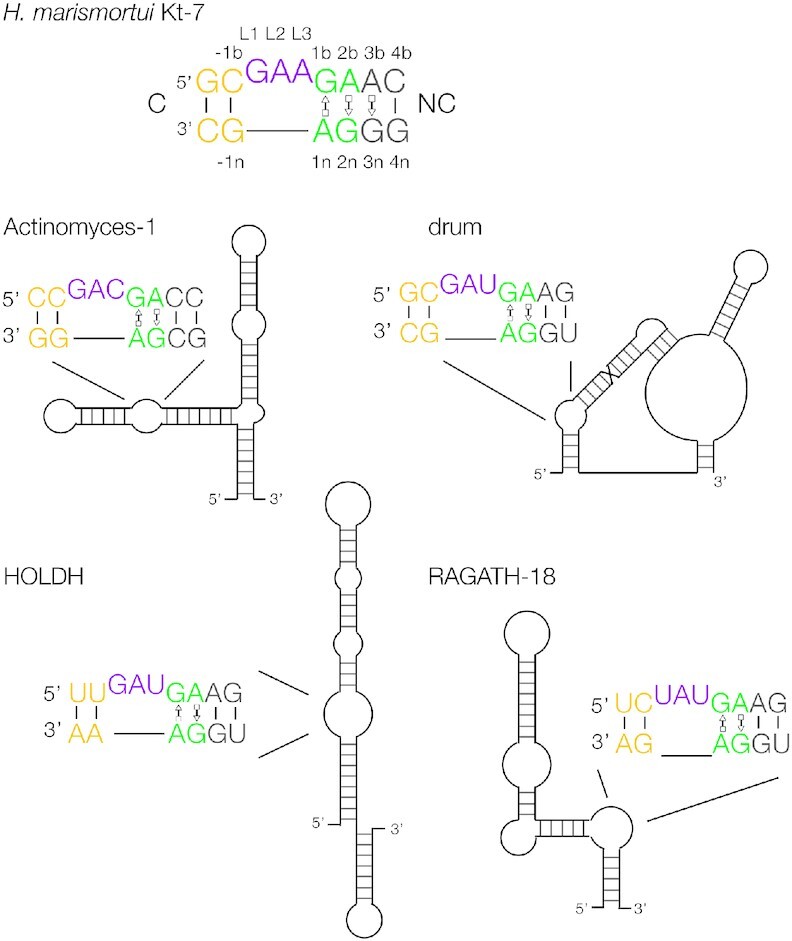
Schematic showing four structured RNA species identified by Weinberg *et al.* (1), with their putative k-turn sequences.

Weinberg *et al.* (21,22) have developed powerful comparative genomic analysis to search for regions of conserved secondary structures of RNA within non-coding intergenic regions. These have been applied to several lineages of bacterial, archaeal and metagenomic sequences. More recently they have applied this approach to selected sub-sets of intergenic regions in order to identify rare structured non-coding RNA species, and so identified 224 novel RNAs (1). The challenge is thus to predict structures of these RNA species, and perhaps suggest their function. We carefully inspected these sequences and identified four probable k-turns and one k-junction. The four putative k-turns were identified in the RNA motifs Actinomyces-1, drum, HOLDH and RAGATH-18 (Figure 2). In addition, we have identified a probable k-junction (23) in a three-way helical junction in the sequence DUF3268 (Supplementary Figure S1). We applied our rules for folding and conformation to the four k-turns. These predicted properties were published before these experiments were initiated (11), and are summarized in Table 1. All four were expected to fold on addition of metal ions, although we could not be sure about HOLDH as it has −1b:−1n = U:A and this had not been studied previously. Three of the four were predicted to adopt an N3 conformation as they each conform to 3b:3n = A:G, but in Actinomyces-1 3b:3n = C:C which predicts an N1 conformation (18). We have therefore studied the folding properties and conformation of these k-turns, finding that application of the rules correctly predicts the experimental properties.

**Table 1.** Key sequences and predicted folding properties and conformations of the four k-turns

k-turn	−1b:−1n	3b:3n	4b:4n	Folding in ions	Conformation N3 or N1
Actinomyces-1	C:G	C:C	C:G	yes	N1
Drum	C:G	A:G	G:U	yes	N3
HOLDH	U:A	A:G	G:U	weak ?	N3
RAGATH-18	C:G	A:G	G:U	yes	N3

## MATERIALS AND METHODS

### RNA synthesis

RNA oligonucleotides were synthesized using ***t***-BDMS phosphoramidite chemistry (24) as described in Wilson *et al.* (25), implemented on an Applied Biosystems 394DNA/RNA synthesizer. RNA was synthesized using ribonucleotide phosphoramidites with 2′*O*-*tert*-butyldimethyl-silyl (*t*-BDMS) protection (26,27) (Link Technologies). All oligoribonucleotides were redissolved in 100 μl of anhydrous DMSO and 125 μl triethylamine trihydrofluoride (Sigma-Aldrich) to remove *t*-BDMS groups, and agitated at 65°C in the dark for 2.5 h. After cooling on ice for 10 min, the RNA was precipitated with 1 ml of butanol, washed once with 70% ethanol and suspended in double-distilled water. Fluorescein (Link Technologies) and Cy3 (GE Healthcare) were attached to the 5′ termini of the oligonucleotides as phosphoramidites in the final cycle of the synthesis, as required.

### RNA purification and hybridization of RNA for fluorescence

RNA was purified by gel electrophoresis in 16% polyacrylamide in 90 mM Tris−borate (pH 8.3), 10 mM EDTA, 7 M urea at 23 W constant power at room temperature. RNA-containing bands were excised and recovered by elution from the crushed fragments. The eluted RNA was precipitated with ethanol and dissolved in 0.1 M TEAA. Fluorescent RNA strands were purified by reversed-phase chromatography on a C_18_ column using an 0.1 M TEAA/acetonitrile gradient. Strands were then hybridized in 50 mM Tris−HCl (pH 7.5), 25 mM NaCl by slowly cooling an equimolar mixture of bulged and unbulged strands from 80°C to 4°C, and purified by gel electrophoresis in 12% polyacrylamide in 45 mM Tris−borate (pH 8.3), 25 mM NaCl buffer at 4°C. The hybridized species were recovered by excising the bands and elution from the crushed fragments followed by precipitation in ethanol. The sequences are listed in full in Supplementary Table S1.

### RNA purification and hybridization of RAGATH-18 k-turn RNA for crystallography

The oligonucleotide 5′-GUCUAUGAAGGCUGGAGAC-3′ from *Clostridium perfringens* was synthesized and then purified by gel electrophoresis in polyacrylamide under denaturing conditions in the presence of 7 M urea. The full-length RNA product was visualized by UV shadowing. The band was excised and electroeluted using an Elutrap Electroelution System (GE Healthcare) into 45 mM Tris−borate (pH 8.5), 5 mM EDTA buffer for 12 h at 150 V at 4°C. The RNA was precipitated with isopropanol, washed once with 70% ethanol, and dissolved in water. The sequence is shown in duplex form in Supplementary Table S2.

### Cloning and transcription of the SAM-I riboswitch containing the Actinomyces-I k-turn

A plasmid containing a gene for the *Thermoanaerobacter tengcongensis* SAM-I riboswitch was changed by site-directed mutagenesis in order to convert the sequence of the k-turn region to that of the Actinomyces-I k-turn. The sequence of the modified plasmid was verified by DNA sequencing, and the sequence of the Actinomyces-I k-turn as inserted into the riboswitch is shown in Supplementary Table S2. The SAM-I riboswitch variant was transcribed from PCR-amplified templates using T7-RNA polymerase. Transcribed RNA was purified by electrophoresis in 12% polyacrylamide gels in 90 mM Tris−borate (pH 8.3), 10 mM EDTA, 7 M urea.

### L7Ae expression and purification

*A. fulgidus* L7Ae cloned into a modified pET-Duet1 plasmid (Novagen) (28) was expressed as a hexahistidine fusion protein in *Escherichia coli* BL21-Gold (DE3) pLysS cells (Stratagene), and purified as reported previously (29).

### FRET analysis of k-turn folding

FRET efficiency was measured from a series of RNA duplex species (Supplementary Table S2), terminally 5′-labeled with fluorescein on the bulged strand and Cy3 on the non-bulged strand, containing central k-turn sequences.

Fluorescein-Cy3-labeled RNA samples were dissolved in 100 μl 90 mM Tris−borate (pH 8.3) and absorption spectra were measured for each RNA between 400 and 600 nm using a Thermo Evolution 201 UV−visible spectrophotometer. Spectra were deconvoluted using a corresponding RNA species labeled only with Cy3, and fluorophore absorption ratios required for FRET analysis were calculated using a program implemented in MATLAB. Fluorescence spectra were recorded in 90 mM Tris-borate (pH 8.3) at 4°C using an SLM-Aminco 8100 fluorimeter. Spectra were corrected for lamp fluctuations and instrumental variations, and polarization artifacts were avoided by setting excitation and emission polarizers crossed at 54.7°. Values of FRET efficiency (*E*_FRET_) were measured using the acceptor normalization method (30) implemented in MATLAB. *E*_FRET_ as a function of Mg^2+^ ion concentration was analyzed on the basis of a model in which the fraction of folded molecules corresponds to a simple two-state model for ion-induced folding, i.e.

(1) $$\begin{eqnarray*}{E_{{\rm{FRET}}}} = {E_0} + \Delta {E_{{\rm{FRET}}}}.{K_A}\left[ {\rm Mg^{2+}} \right]{\rm{ }}/{\rm{ }}\left( {1{\rm{ }} + {K_A}\left[ {\rm Mg^{2+}} \right]} \right)\nonumber\\ \end{eqnarray*}$$

where *E*_0_ is the FRET efficiency of the RNA in the absence of added metal ions, Δ*E*_FRET_ is the increase in FRET efficiency at saturating metal ion concentration, [Mg^2+^] is the prevailing Mg^2+^ ion concentration and *K*_A_ is the apparent association constant for metal ion binding. Data were fitted to this equation by nonlinear regression.

### Crystallization, structure determination and refinement

#### The RAGATH k-turn as simple duplex

Crystals grew in space group *I*222 with unit cell dimensions *a* = 62.8 Å, *b* = 74.9 Å and *c* = 136.6 Å. Diffraction data were collected on beamline I04-1 at the Diamond Light Sources.

From crystal density considerations, five RNA molecules were expected to be present in the asymmetric unit. The structure was determined by molecular replacement using the program PHASER (31) with *Hm*Kt-7 (PDB 4CS1) as the search model.

#### Actinomyces-1 k-turn in the SAM-I riboswitch

The SAM-I riboswitch variants were crystallized using the hanging drop method. Drops were seeded using a micro-crystals taken from crystal trays containing the unmodified RNA (corresponding to structure PDB 4B5R). Crystals of space group *P*4_3_2_1_2 were obtained. Diffraction data were collected on different beamlines, including BL17U, BL18U1 and BL19U1 at SSRF. Data were indexed, integrated and scaled using iMOSFLM and Scala from the CCP4 suite of programs (32,33). Structures were solved by performing molecular replacement using PDB entry 3GX5 (34) or 5FK3 as a preliminary model. The structures were refined using Phenix refine, and the model was built using COOT (35). A composite omit map was calculated using Phenix (36).

Crystallographic data and statistics are presented in Supplementary Table S3.

## RESULTS

### Ion-induced folding of a standard k-turn depends on its −1b:−1n sequence

*H. marismortui* Kt-7 (*Hm*Kt-7) is the best characterized k-turn structure (11). In free solution it folds into the k-turn conformation on addition of divalent metal ions, or the binding of *A. fulgidus* L7Ae (*Af*L7Ae) protein. 17 independent crystal structures in a variety of environments reveal that when not *in situ* in the 50S ribosomal subunit *Hm*Kt-7 adopts the N3-conformation (18). We have found previously that the base pair at the 3b:3n position is an important determinant of the ability of the RNA to fold into the k-turn conformation on addition of metal ions (12), as well as the N3- or N1-conformation adopted (18). From these studies we deduced a set of rules associating the 3b:3n sequence, the folding properties and the conformation adopted (Figure 1). We have also found that the −1b:−1n sequence also has a strong influence on the ability of k-turns to fold on addition of metal ions (17). However, our previous studies were limited to the analysis of folding in k-turns with −1b:−1n = G:C or C:G. We therefore studied ion-induced folding using fluorescence resonance energy transfer (FRET) in a sequence background of *Hm*Kt-7, where the −1b:−1n was one of the four possible Watson−Crick base pairs. Each of the Kt-7 variants with the different −1b:−1n sequences had 3b:3n = A:G that is known to support ion-induced folding (12).

**Figure 3.**
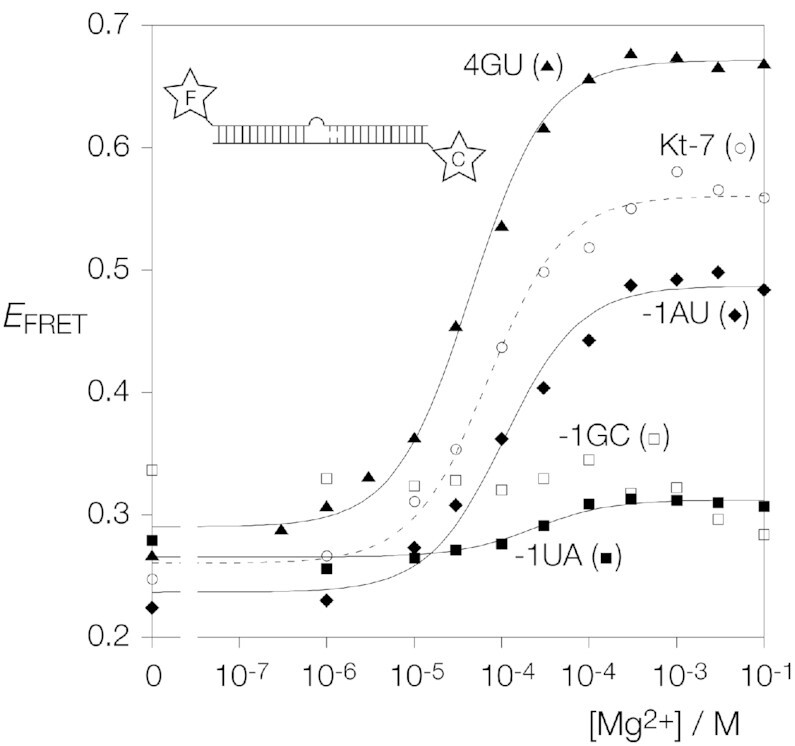
Mg^2+^ ion-induced folding of Kt-7 variants studied by steady-state FRET. Each species was studied as an equivalent 25 bp duplex with a central Kt-7 k-turn with or without a single base pair change in the -1b:-1n or 4b:4n position with fluorescein (donor) and Cy-3 (acceptor) attached at the 5′ termini. FRET efficiency (*E*_FRET_) was measured by the acceptor ratio method (30). *E*_FRET_ is plotted as a function of Mg^2+^ ion concentration for each species, and fitted to a two-state binding model. Folding of the k-turn kinks the axis of the RNA, so bringing the fluorophores closer together and increasing *E*_FRET_. Changing the -1b:-1n base pair has a significant effect on folding, in the order C:G (the natural sequence; open circle symbols) > A:U (filled diamond symbols) > U:A (filled square symbols, weak folding only) > G:C (open square symbols; no folding detected for this species). Changing 4b:4n in Kt-7 (i.e. with the natural −1b:−1n = C:G) led to significantly better folding (filled triangle symbols).

We prepared the four 25 bp RNA duplex species with central *Hm*Kt-7 k-turns with −1b:−1n = C:G (wild type), C:G, U:A or A:U (Supplementary Table S1). These were fluorescently labeled on the 5′ termini with fluorescein (bulged strand) and Cy-3 (non-bulged strand). Fluorescence spectra were recorded with excitation of fluorescein and Cy3 as a function of added MgCl_2_ concentration, and FRET efficiency (*E*_FRET_) measured using the acceptor ratio method (30). The data are plotted in Figure 3 and fitted to a two-state binding model for ion-induced folding (Supplementary Table S4). As expected, the standard *Hm*Kt-7 (−1b:−1n = C:G) folds on addition of Mg^2+^ ions, changing from *E*_FRET_ = 0.25 to 0.56 as the RNA kinking concomitant with k-turn conformation brings the fluorophores closer together, with a calculated apparent *K*_d_ = 70 μM. As observed previously, addition of *Af*L7Ae increased *E*_FRET_ slightly further to 0.63. Reversing the −1b:−1n base pair to G:C resulted in an RNA that underwent no folding into the k-turn conformation, either with addition of Mg^2+^ ions or *Af*L7Ae protein. We have previously found that -1b:-1n = G:C prevents ion-induced folding in similar sequence backgrounds, and noted that natural k-turns have a strong preference for −1b:−1n = C:G (17). The folding of *Hm*Kt-7 variants with −1b:−1n = U:A and A:U have not been analyzed previously. From the new data we observe that *Hm*Kt-7 with −1b:−1n = A:U folds almost as well as wild-type Kt-7 on addition of Mg^2+^ ions, although a slightly higher concentration of Mg^2+^ is required, *K*_d_ = 106 μM. Addition of Mg^2+^ ions to *Hm*Kt-7 with −1b:−1n = U:A led to a much smaller increase in *E*_FRET_ to 0.3, although a folding transition was apparent. This required a significantly higher concentration of Mg^2+^ ions, with an apparent *K*_d_ = 231 μM.

In summary we see that the −1b:−1n sequence has a significant effect on the folding of the k-turn. −1b:−1n = C:G or A:U fold well on addition of Mg^2+^ ions, while 1b:−1n = G:C is unable to fold at all. −1b:-1n = U:A only folds weakly in the Kt-7 context, and folding ability may then depend upon other sequence elements in the k-turn. We can thus expand our sequence rules for k-turns to include all the −1b:−1n base pairs, summarized in Figure 1.

### Ion-induced folding of *Hm*Kt-7 with a G:U base pair substituted at the 4b:4n sequence

Inspection of the four putative k-turn sequences shows that for three of them 3b:3n = A:G (as in the archetypal k-turn *Hm*Kt-7) and 4b:4n = G:U. We have previously observed that an adjacent G:U base pair can have a major structural effect on an A:G base pair (20), and this suggests that placing G:U in the 4b:4n position might affect the properties of a k-turn. We therefore constructed a variant of *Hm*Kt-7 equivalent to the fluorescein-Cy3 duplexes above, but with 4b:4n = G:U in place of C:G (Supplementary Table S1), and studied its ion-induced folding properties by steady-state FRET. *E*_FRET_ was measured as a function of magnesium ion concentration. The titration data are plotted in Figure 3 and fitted to a two-state binding model of ion-induced folding (Supplementary Table S4). The folding isotherm for the Kt-7 with 4b:4n = G:U differs from that of the unmodified Kt-7 in two respects. The limiting value of *E*_FRET_ achieved is 0.67 for the variant with 4b:4n = G:U, compared to 0.56 for the unmodified Kt-7. Thus, the population is more completely folded with G:U in the 4b:4n position. Secondly, fitting the data gives an apparent *K*_d_ of 44 μM for the variant compared to 70 μM for *Hm*Kt-7. Thus the effect of changing 4b:4n to G:U in the context of *Hm*Kt-7 is to fold more completely, occurring at a lower magnesium ion concentration.

### Ion-induced folding properties of the four predicted k-turn structures

The key sequences and predicted folding properties and conformations of the four k-turns are summarized in Table 1. All have 3b:3n = A:G or C:C which are known to support ion-induced folding. Three have −1b:−1n = C:G which also supports ion-induced folding, but HOLDH has −1b:−1n = U:A which leads to weak folding of Kt-7 in response to metal ions. Application of the rules therefore suggests that Actinomyces-1, drum and RAGATH-18 should fold on addition of Mg^2+^ ions, and HOLDH would fold more weakly. We have therefore used steady-state FRET analysis to examine the potential folding of these four RNA species.

### Steady-state FRET analysis of ion-induced folding properties of the four predicted k-turn structures

**Figure 4.**
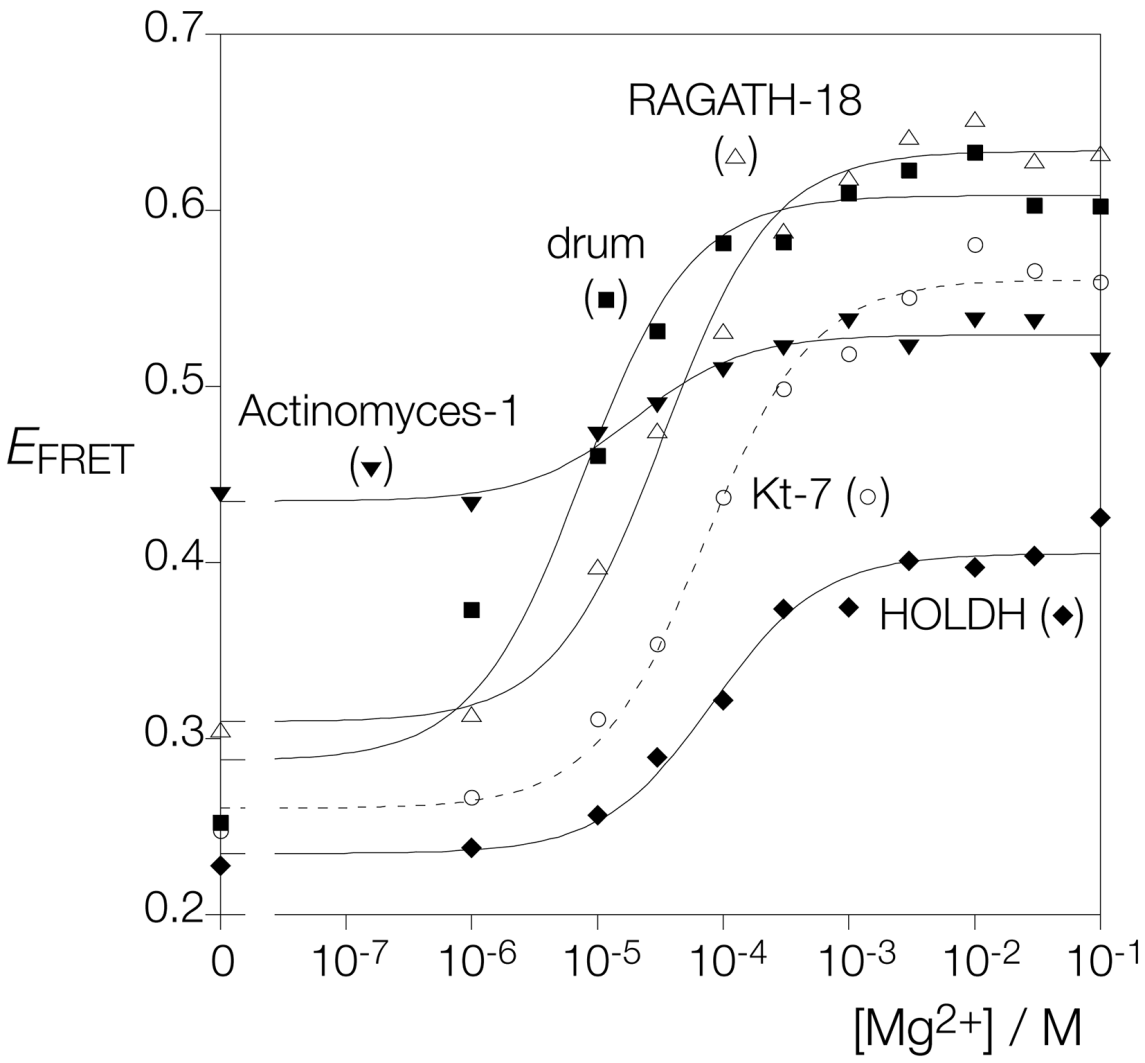
Mg^2+^ ion-induced folding the four Weinberg-Breaker k-turns studied by steady-state FRET. *E*_FRET_ was measured by the acceptor ratio method (30) and plotted as a function of Mg^2+^ ion concentration for each species. The data have been fitted to a two-state binding model. The corresponding data for unmodified *Hm*Kt-7 are plotted on the same axes for reference (open circle symbols and the fit shown by the broken line). RAGATH-18 (open triangle symbols) and drum (filled square symbols) are well folded by the addition of Mg^2+^ ions, achieving a higher eventual *E*_FRET_ than Kt-7. HOLDH (filled diamond symbols) is less well folded. Actinomyces-I (filled inverted diamond symbols) begins at a higher value of *E*_FRET_ but achieves a very similar level of folding to Kt-7 at higher Mg^2+^ ion concentrations.

We prepared four fluorescein-Cy-3-labeled 25 bp RNA duplex species with central k-turns corresponding to the Actinomyces-1, drum, HOLDH and RAGATH-18 sequences (Supplementary Table S1). In each case, the k-turn has three base pairs of the C-helix, the loop, and four base pairs of the NC helix including the G:A and A:G base pairs and any G:U base pairs within. Fluorescence spectra were recorded as above as a function of added MgCl_2_ concentration, and FRET efficiency (*E*_FRET_) measured using the acceptor ratio method (30). MgCl_2_ titration data are plotted in Figure 4 and fitted to a two-state binding model of ion-induced folding. The resulting *E*_FRET_ and apparent Mg^2+^ binding affinities are summarized in Supplementary Table S4.

All four sequences fold into a state with elevated FRET efficiency on addition of Mg^2+^ ions. drum and RAGATH-18 achieve a final *E*_FRET_ close to 0.6. Their folding isotherms are similar to those of *Hm*Kt-7, although they fold at lower ionic concentrations with *K*_d_ = 8 μM and 33 μM respectively. Both sequences conform to −1b:−1n = C:G and 3b:3n = A:G, so were predicted to be well-folding k-turns. Their lower apparent ion binding affinities must reflect other aspects of their sequence. The L1 and L2 positions (that stack on the ends of the C and NC helices respectively) might exert some influence, but drum and *Hm*Kt-7 share a GA sequence. Given that placing a G:U base pair in the 4b:4n position of Kt-7 results in better ion-induced folding it is probable that this is similarly responsible for the superior folding of the RAGATH-18 and drum k-turns. The HOLDH sequence has 3b:3n = A:G, but −1b:−1n = U:A. As we have shown above, inclusion of the latter feature leads to weak ion-induced folding in *Hm*Kt-7. The folding of HOLDH induced by addition of Mg^2+^ ions is clearly weaker than that of drum and RAGATH-18, reaching a limiting *E*_FRET_ = 0.4. Yet that is rather better than the folding of the variant of *Hm*Kt-7 with −1b:−1n = U:A, likely a consequence of the 4b:4n = G:U. The 4b:4n = G:U provides a partial rescue of the effect of the −1b:−1n = U:A.

The Actinomyces-1 sequence has −1b:−1n = C:G and 3b:3n = C:C, and was therefore predicted to undergo folding into the k-turn conformation on addition of Mg^2+^ ions. The RNA underwent folding up to a limiting *E*_FRET_ of 0.52, similar to *Hm*Kt-7. The main distinction between the behavior of the Actinomyces-1 RNA and the other three k-turns (plus *Hm*Kt-7) is the high value of *E*_FRET_ in the absence of added Mg^2+^ ions. This indicates that the structure of this RNA before folding into the k-turn conformation is different. All these molecules likely adopt the structure of a standard 3-nucleotide bulge under these conditions i.e. kinked, but not to the extent observed in a standard k-turn geometry. It appears that the Actinomyces sequence kinks to a greater extent prior to k-turn folding.

Addition of *Af*L7Ae protein to each of the four k-turns led to similar extents of folding, in the range *E*_FRET_ = 0.53 to 0.67, and similar to the value for *Hm*Kt-7 of 0.63. Each therefore binds *Af*L7Ae and probably adopts a very similar conformation.

### The N3 / N1 conformations of the predicted k-turn structures

We have found that the key determinant of the N3 or N1 conformation of a k-turn is the 3b:3n sequence (18). The k-turns drum, HOLDH and RAGATH-18 all have 3b:3n = A:G and are quite similar to the well-characterized *Hm*Kt-7 in sequence. We therefore predicted that these three would adopt the N3 conformation. By contrast, Actinomyces-1 has 3b:3n = C:C which is predicted to confer the N1 conformation according to our conformational rules (18). We therefore set out to determine the structure of the Actinomyces-1 k-turn, and one of the other three by X-ray crystallography. We have determined structures of the RAGATH-18 k-turn as a duplex RNA structure, and the Actinomyces-1 k-turn in the context of the SAM-I riboswitch.

### Conformation of the RAGATH-18 k-turn

**Figure 5.**
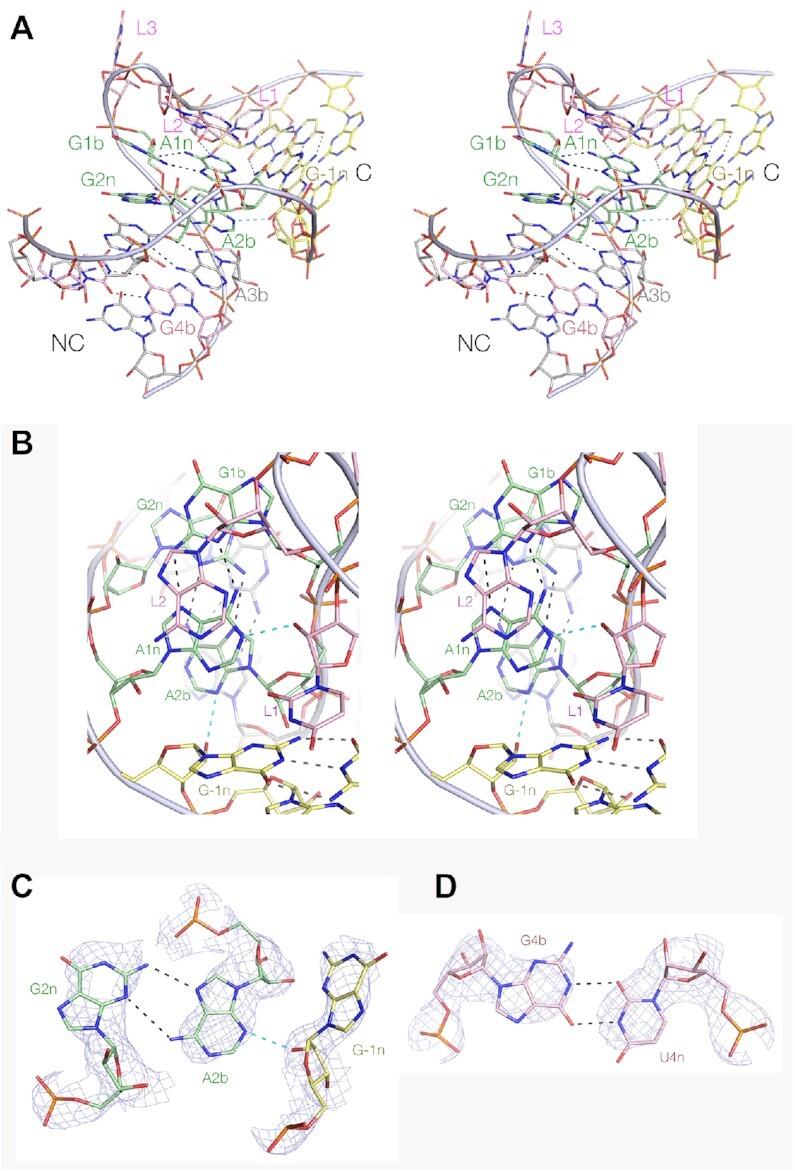
The crystal structure of the RAGATH-18 k-turn. The structure was solved as an RNA duplex containing two k-turns with two-fold symmetry at a resolution of 2.5 Å. (**A**) Parallel-eye stereoscopic view of the overall structure seen from the side of the non-bulged strand, with the NC helix on the left and the C-helix on the right. (**B**) Parallel-eye stereoscopic view down onto the major groove splayed apart on the outside of the k-turn. The key cross-strand hydrogen bonds L1 O2′ to A1n and G-1n O2′ to A2b are highlighted cyan. (**C**) The G2n:A2b:G-1n interaction with the electron density shown. The G-1nO2′ donates its proton to the A2b N3 ring nitrogen atom, and there are two hydrogen bonds between G2n N2 to A2b N7 and A2b N6 to G2n N3. RAGATH-18 is therefore a standard N3 conformation k-turn (15). (**D**) The G4b:U4n base pair with the electron density shown.

The RAGATH-18 RNA was crystallized as a duplex containing two k-turns with two-fold symmetry (Supplementary Table S2). The structure was solved by molecular replacement at a resolution of 2.5 Å (PDB ID 7EAG). The RNA forms a standard k-turn structure, where both the tandem G:A base pairs at the 1b:1n and 2n:2b positions form the usual *trans*-sugar-Hoogsteen pairs (Figure 5). The standard cross-strand hydrogen bonds from the L1 O2′ to A1n N1, and from the -1n O2′ to A2b are formed (Figure 5B). The latter is accepted by A2b N3, and further stabilized by a hydrogen bond between G-1n N2 and A2b O2′ (Figure 5C). Thus the RNA adopts the N3 conformation as predicted. Consistent with this, the A2b:G2n base pair is associated by two hydrogen bonds including that from A2b N6 to G2n N3. This contrasts with the structure adopted by the Actinomyces-1 k-turn described in the following section. The A3b:G3n base pair is also a standard *trans*-sugar-Hoogsteen pair, and the G4b:U4n adopts a *cis*-wobble base pair conformation with two hydrogen bonds between the nucleobases (Figure 5D).

### Conformation of the actinomyces-1 k-turn

**Figure 6.**
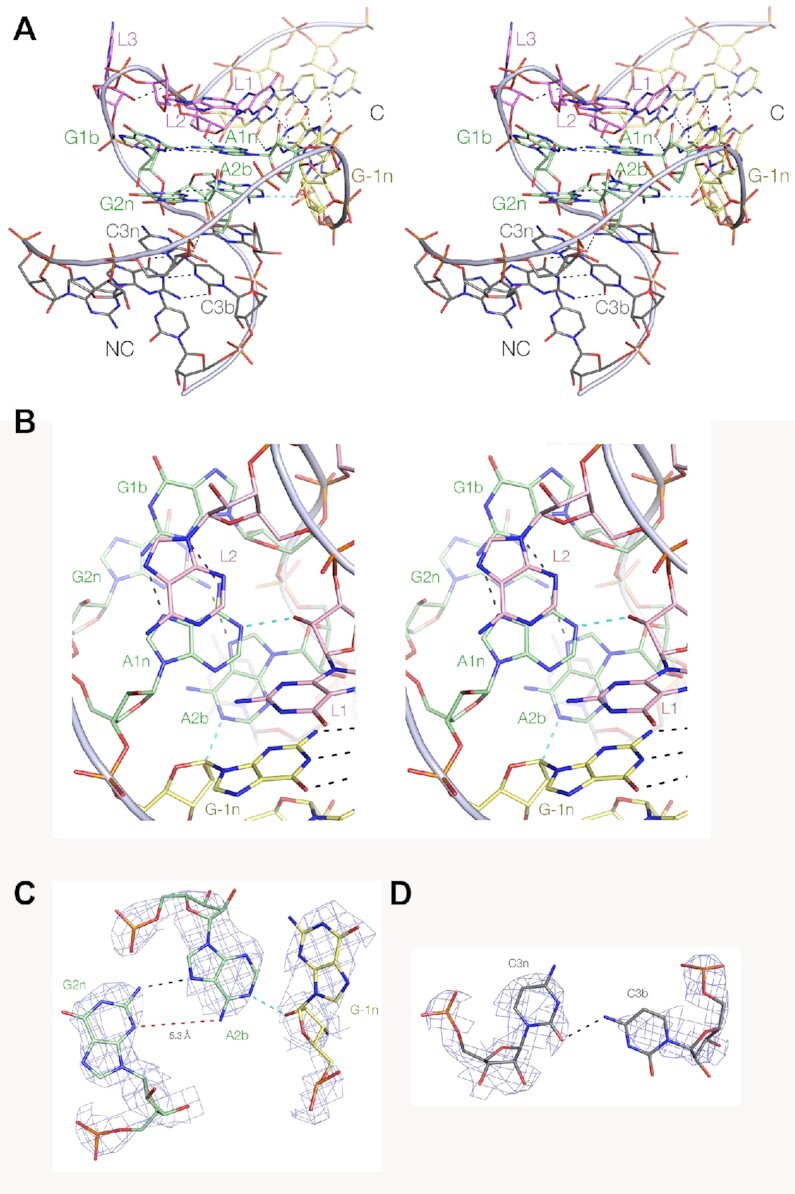
The crystal structure of the Actinomyces-1 k-turn. The k-turn sequence was inserted into the SAM-I riboswitch and structure of the recombinant riboswitch solved at a resolution of 2.85 Å. (**A**) Parallel-eye stereoscopic view of the overall structure seen from the side of the non-bulged strand, with the NC helix on the left and the C-helix on the right. (**B**) Parallel-eye stereoscopic view down onto the major groove splayed apart on the outside of the k-turn. The key cross-strand hydrogen bonds L1 O2′ to A1n and G-1n O2′ to A2b are highlighted cyan. (**C**) The G2n:A2b:G-1n interaction with the electron density shown. The G-1n O2′ donates its proton to the A2b N1 ring nitrogen atom. The A2b nucleobase is rotated around so that the distance between A2b N6 and G2n N3 is lengthened to 5.3 Å (shown in red). The Actinomyces-1 k-turn therefore adopts a standard N1 conformation (15). (**D**) The C3b:C3n base pair with the electron density shown. This forms a *cis* base pair associated by a single hydrogen bond between C3b N4 and C3n O2.

The Actinomyces-1 k-turn sequence was inserted in place of the natural k-turn in the SAM-I riboswitch (Supplementary Table S2). We have previously found that this is a versatile vehicle for crystallizing many different k-turns, and accommodates k-turns in both the N3 and N1 conformation (18). The Actinomyces-1 k-turn-containing riboswitch RNA was transcribed and crystallized, and solved by molecular replacement at a resolution of 2.85 Å (Figure 6, Supplementary Figure S2) (PDB ID 7EAF).

The RNA adopts a standard k-turn geometry, with tandem *trans*-sugar-Hoogsteen G:A base pairs, and with both the cross-strand hydrogen bonds (Figure 6B). However, in this case the hydrogen bond from G-1n O2′ is accepted by A2b N1 (Figure 6C). This requires the A2b nucleobase to rotate in plane so that the A2bN6 – G2nN3 distance is lengthened to 5.3 Å. These features are all characteristic of the N1 conformation. The C:C base pair in the 3b:3n position is connected by a single hydrogen bond between C3bN4 and C3nO2 (Figure 6D).

## DISCUSSION

We now have an expanded set of rules for associating the folding properties and conformation of k-turns with their sequence. In brief:

- The 16 possible 3b:3n sequences are very important determinants of whether or not ion-induced folding occurs, and are the major determinant of the N3/N1 conformation of the k-turn.
- The -1b:-1n sequence is also an important determinant of ion-induced folding, and we now know the effect of each of the four Watson−Crick base pairs in this position.
- A G:U base pair in the 4b:4n position facilitates ion-induced folding, at least for those k-turns for which 3b:3n = A:G.

The crystal structure of the RAGATH-18 k-turn shows that G4b:U4n adopts a standard wobble base pair. We have shown that a G:U base pair can alter the structure of an adjacent G:A base pair (20), but can we suggest a reason for its effect on k-turn folding in metal ions? When we solved a structure of *Hm*Kt-7 at high resolution we found two partially dehydrated magnesium ions interacting with the O6 atoms of G2n and G3n, and sharing one inner sphere water molecule (12). Moreover there was further electron density in that structure indicating that this chain of ions might extend further down the major groove on the outer face of the k-turn. Examination of the RAGATH-18 crystal structure now reveals unassigned electron density in the major groove (Supplementary Figure S3). In addition to *F*_o_− *F*_c_ density close to the terminal phosphates, we observe three peaks in the planes of successive base pairs, close to the Hoogsteen edges of G2n, G3n and to the O4 and O6 atoms of U4n and G4b respectively. U4n O4 is prominent in the major groove because of the wobble conformation of the base pair. This local conformation may stabilize a chain of bound metal ions, providing a plausible explanation for the enhanced folding of k-turns with 4b:4n = G:U.

The expanded set of rules for ion-induced folding and conformation has been applied to the four sequences identified in the 224 structured RNA Weinberg-Breaker species. All four clearly fold into a k-turn conformation, and all fold on addition of magnesium ions alone as predicted. The folding of the HOLDH k-turn is clearly hindered by the U:A base pair in the −1b:−2n position, but alleviated to a significant degree by the G:U base pair in the 4b:4n position. The folding of the drum and RAGATH-18 k-turns achieves a higher extent and occurs at a lower magnesium ion concentration than *Hm*Kt-7, again showing the effect of the G4b:U4n base pair. Application of the conformational rules predicted that the drum, HOLDH and RAGATH-18 would each fold into the N3 conformation, while the Actinomyces-1 k-turn should adopt the N1 conformation. X-ray crystallography of the RAGATH-18 and Actinomyces-1 k-turns confirm that both form standard k-turn structures, with N3 and N1 conformations respectively. Thus the folding and conformational properties of these unknown k-turns all conform to our rules. Thus far these rules have proved to be robust. However it is conceivable that exceptions could exist, and we do not know if they will apply to k-junctions for example. But to date they have passed every test, and can now be applied to k-turn sequences of unknown properties.

## DATA AVAILABILITY

Atomic coordinates have been deposited in the Protein Data Base with identifiers 7EAF (Actinomyces k-turn) and 7EAG (RAGATH-18 k-turn).

## Supplementary Material

gkab333_Supplemental_File
