# Supplementary material for: Structure and folding of four putative kink turns identified in structured RNA species in a test of structural prediction rules
Source: Nucleic Acids Res. 2021 May 12;49(10):5916–24. doi: 10.1093/nar/gkab333 (PMC8191799; doi:10.1093/nar/gkab333)
Supplement: gkab333_Supplemental_File [file gkab333_supplemental_file.pdf]

Structure and folding of four putative kink turns identified in structured RNA species in a test of structural prediction rules

Lin Huang, Xinli Liao, Mengxiao Li, Jia Wang, Xuemei Peng, Timothy J. Wilson, David M.J. Lilley

SUPPLEMENTARY MATERIALS

SUPPLEMENTARY FIGURES

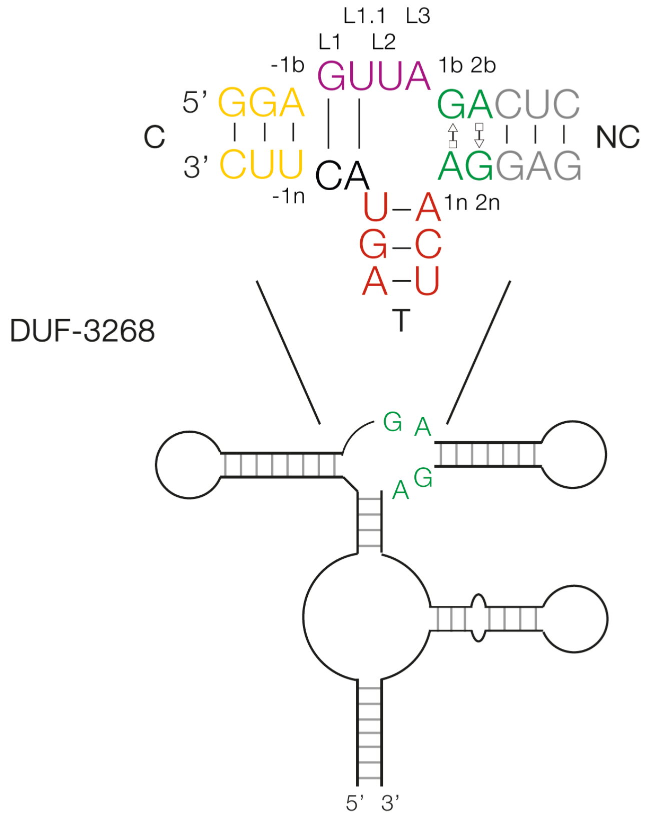

Figure S1. A possible k-junction in the Weinberg-Breaker sequences. The three-way junction in DUF3268 conforms to all the features of a putative k-junction, and the nucleotide positions are labelled as in previous work (1).

1. Wang, J., Daldrop, P., Huang, L. and Lilley, D.M. (2014) The k-junction motif in RNA structure. *Nucleic Acids res*., **42**, 5322-5331.

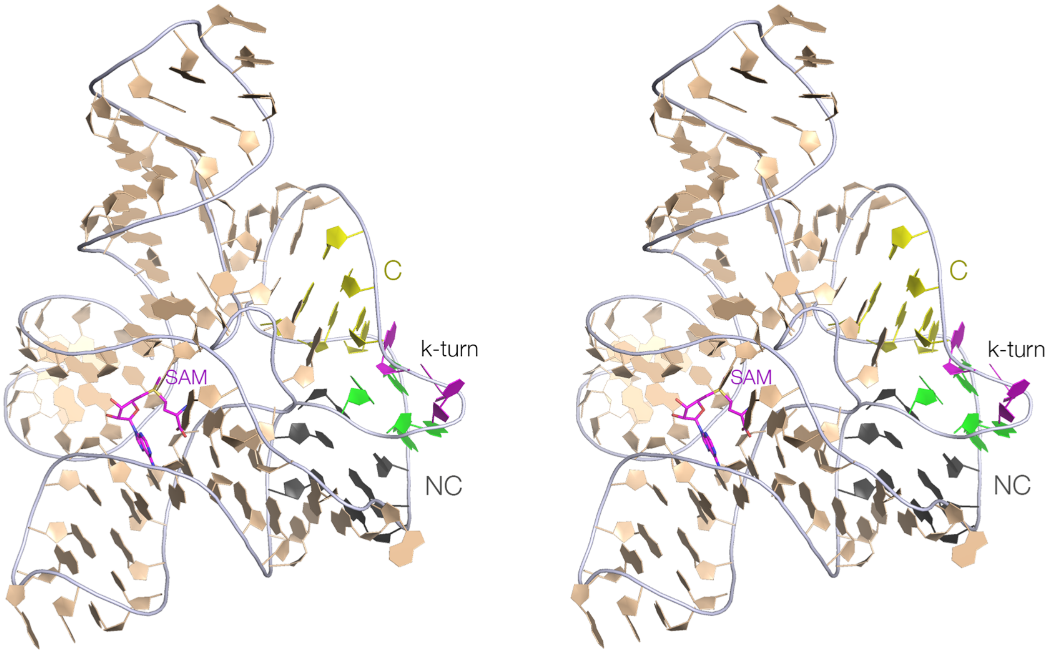

Figure S2. Parallel-eye stereoscopic image of the crystal structure of the SAM-I riboswitch in which the Actinomyces-I k-turn has replaced the natural riboswitch k-turn. The k-turn is shown in our standard coloring, and the rest of the riboswitch in straw color. The SAM ligand is shown in stick form, colored magenta.

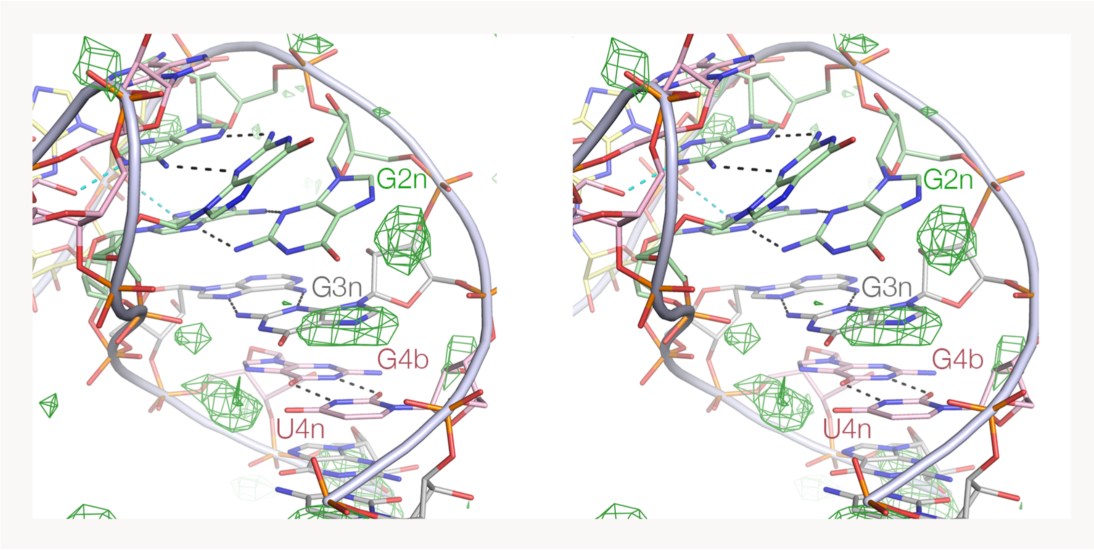

Figure S3. Unassigned electron density in the major groove of RAGATH-18 that could correspond to hydrated metal ions. Parallel-eye stereoscopic image of the NC helix of the k-turn viewed into the major groove. The **Fo-Fc** electron density map is colored green. Unassigned electron density is observed adjacent to the major groove edges of the nucleobases of G2n, G3n and U4n. These could correspond to bound metal ions.

SUPPLEMENTARY TABLES

Kt7 variant sequences :

*Hm*Kt-7 -1b:-1n = C:G (i.e. natural sequence *Hm*Kt-7)
F-CCAGUCAGUGGC^{GAA}GAACCAUGUCAGG
GGUCAGUCACCG------AGGGGUACAGUCC-Cy

*Hm*Kt-7 -1b:-1n = G:C
F-CCAGUCAGUGGG^{GAA}GAACCAUGUCAGG
GGUCAGUCACCC------AGGGGUACAGUCC-Cy

*Hm*Kt-7 -1b:-1n = U:A
F-CCAGUCAGUGGU^{GAA}GAACCAUGUCAGG
GGUCAGUCACCA------AGGGGUACAGUCC-Cy

*Hm*Kt-7 -1b:-1n = A:U
F-CCAGUCAGUGGA^{GAA}GAACCAUGUCAGG
GGUCAGUCACCU------AGGGGUACAGUCC-Cy

*Hm*Kt-7 4b:4n = G:U
F-CCAGUCAGUGGC^{GAA}GAAGCAUGUCAGG
GGUCAGUCACCG-----AGGUGUACAGUCC-Cy

Weinberg-Breaker k-turn sequences :

Actinomyces-1
F-CCAGUCAGUCCC^{GAC}GACCCAUGUCAGG
GGUCAGUCAGGG-----AGCGGUACAGUCC-Cy

drum
F-CCAGUCAGUGGC^{GAU}GAAGCAUGUCAGG
GGUCAGUCACCG------AGGUGUACAGUCC-Cy

HOLDH
F-CCAGUCAGUGUU^{GAU}GAAGCAUGUCAGG
GGUCAGUCACAA------AGGUGUACAGUCC-Cy

RAGATH-18
F-CCAGUCAGUUUC^{UAU}GAAGCAUGUCAGG
GGUCAGUCAGAG-----AGGUGUACAGUCC-Cy

Table S1. RNA sequences used in fluorescence experiments. All k-turn constructs were fluorescently labelled with fluorescein at the 5’-terminus of the bulged strand (F) and with Cy-3 at the 5’-terminus of the non-bulged strand (Cy). The key sequence differences for the *Hm*Kt-7 variants are highlighted yellow. All sequences are written 5’ to 3’.

RAGATH-18

GUC^{UAU}GAAGGCUGGA-----GAC
CAG-----AGGUCGGAGA$_{UAU}$CUG

Actinomyces-1

CC^{GAC}GACC
GG-----AGCG

Table S2. Sequence of the RNA species containing the RAGATH-18 and Actinomyces-1 k-turns used for X-ray crystallography. The RAGATH-18 was synthesized as a self-complementary strand that forms two k-turns with an overall 2-fold symmetry. The Actinomyces-1 k-turn was generated in the SAM-I riboswitch by mutagenesis of the *T. tengcongensis* SAM-I riboswitch gene. All sequences are written 5’ to 3’.

Name	RAGATH-18 k-turn	Actinomyces-1 k-turn
PDB	7EAG	7EAF
**Data collection**		
Space group	I222	$P4_32_12$
Cell dimensions		
a, *b*, *c* (Å)	62.8, 74.9, 136.6	61.5, 61.5, 157.9
α, β, γ (°)	90 90 90	90 90 90
Wavelength	0.9119	0.9785
Resolution (Å)	65.70 – 2.40 (2.44 – 2.40)	30.00 – 2.80 (2.95 – 2.80)
R_{merge}	0.044 (1.691)	0.143 (1.751)
$I / \sigma I$	20.2 (0.3)	8.9 (1.4)
CC (1/2)	0.99 (0.67)	0.99 (0.77)
Completeness (%)	99.9 (100)	99.6 (99.6)
Redundancy	6.3 (6.4)	7.2 (7.7)
**Refinement**		
Resolution (Å)	65.68 – 2.50 (2.59 – 2.50)	28.64 – 2.85 (2.95 – 2.85)
No. reflections	11491 (1127)	7538 (739)
R_{work} / R_{free}	0.218 / 0.273	0.263 / 0.296
No. atoms		
macromolecules	2040	2048
ligands		34
solvent		5
B-factors		
macromolecules	120.9	75.2
ligands		71.4
solvent		59.8
R.m.s. deviations		
Bond lengths (Å)	0.002	0.004
Bond angles (°)	0.59	1.97

*Values in parentheses are for highest-resolution shell.

Table S3. Details of data collection and refinement statistics for the crystallographic data as deposited with the PDB.

k-turn	-1b:-1n	3b:3n	4b:4n	E_{FRET} final	K_d^{app} Mg^{2+} / μM	E_{FRET} L7Ae
Kt-7 -1CG	C:G	A:G	G:C	0.56	70	0.70
Kt-7 -1AU	A:U	A:G	G:C	0.49	106	0.68
Kt-7 -1UA	U:A	A:G	G:C	0.31	231	0.64
Kt-7 -1GC	G:C	A:G	G:C	no folding		
Kt-7 4GU	C:G	A:G	G:U	0.67	44	0.71
Actinomyces-1	C:G	C:C	C:G	0.53	20	0.66
drum	C:G	A:G	G:U	0.61	8	0.71
HOLDH	U:A	A:G	G:U	0.41	82	0.64
RAGATH-18	C:G	A:G	G:U	0.63	33	0.68

Table S4. Folding characteristics of k-turns derived from steady-state FRET titration by magnesium ions. The final E_{FRET} values and apparent affinity for magnesium ions (presented as dissociation constants K_d) were calculated from the fits to the titration curves as shown in figures 3 and 4 of the main text. After titration with magnesium ions 100 nM *A. fulgidus* L7Ae was added, and the E_{FRET} values measured are presented in the final column.
